# Diabetes Mellitus and Gynecological and Inflammation Disorders Increased the Risk of Pregnancy Loss in a Population Study

**DOI:** 10.3390/life14070903

**Published:** 2024-07-19

**Authors:** Chun-Gu Cheng, Sheng-Hua Su, Wu-Chien Chien, Ryan Chen, Chi-Hsiang Chung, Chun-An Cheng

**Affiliations:** 1 Department of Emergency Medicine, Taoyuan Armed Forces General Hospital, Taoyuan 32549, Taiwan; doc50015@yahoo.com.tw; 2 Department of Emergency Medicine, Tri-Service General Hospital, National Defense Medical Center, Taipei 11490, Taiwan; 3 Department of Pulmonary Medicine, Cheng-Hsin General Hospital, Taipei 11220, Taiwan; chgh9241@gmail.com; 4 School of Public Health, National Defense Medical Center, Taipei 11490, Taiwan; 5 Department of Medical Research, Tri-Service General Hospital, National Defense Medical Center, Taipei 11490, Taiwan; 6 Upper School, Taipei American School, Taipei 111039, Taiwan; 666rcthepro@gmail.com; 7 Department of Neurology, Tri-Service General Hospital, National Defense Medical Center, Taipei 11490, Taiwan

**Keywords:** diabetes mellitus, pregnancy loss, risk

## Abstract

(1) Background: Diabetes mellitus (DM) induces oxidative stress and inflammation with negative effect on pregnancy outcomes. This study aimed to determine whether DM increases the risk of pregnancy loss and to identify other potential risk factors; (2) Methods: We identified female patients diagnosed with DM from 2000–2015 in the Taiwanese National Health Insurance Research Database according to the *International Classification of Diseases*, Ninth Edition, Clinical Modification (ICD-9 CM) code 250. The event was pregnancy loss, defined as ICD-9 CM codes 630–639, which was tracked until 31 December 2015. The control group included 4-fold more non-DM female patients who were matched for age and disease severity. Multivariate Cox regression was employed to determine the risk factors associated with pregnancy loss; (3) Results: The hazard ratio (HR) for the risk of pregnancy loss due to DM was 1.407 (95% confidence interval: 1.099–1.801, *p* = 0.007), and the risk factors for older age, gynecological disorders and inflammation disorders were included. (4) Conclusions: The study concluded that women with DM have a greater risk of experiencing pregnancy loss. Healthcare providers should proactively manage and educate diabetic patients to reduce their risk of pregnancy loss. Understanding other probable risk factors can help in developing targeted interventions and support systems for women to improve pregnancy outcomes.

## 1. Introduction

Pregnancy loss is a non-viable pregnancy, defined as the natural termination of a pregnancy before the fetus is viable outside the uterus, and remains a significant clinical concern with complex etiological factors [1]. It is a complex process with various potential underlying mechanisms, including genetic abnormalities, uterine anatomical abnormalities, progesterone deficiency [2], immunological factors, and environmental factors, such as infections or toxicity [1].

There were more pregnancy outcomes in the Asian group. with a 5-fold greater risk of poor pregnancy outcomes, including abortion and malformation, in pregnant Asian women with diabetes mellitus (DM) than in pregnant Caucasian women in a study conducted in England [3]. A previous study reported an odds ratio (OR) of 1.11 for the risk of pregnancy loss in mothers with higher fasting blood sugar than in mothers with normal fasting blood sugar among pregnant women in China [4]. Compared with all live births in the USA, women with recurrent pregnancy loss are associated with prediabetes, with an adjusted OR of 1.9 [5]. Previous studies have indicated an increased risk of pregnancy loss in diabetic women, highlighting the multifaceted interplay among metabolic dysfunction, endocrine abnormalities, oxidative stress, and placental impairment [6].

Successful pregnancy relies on immune adaptations that allow fetal survival and development while protecting the mother. The initial phase of pregnancy is characterized by an inflammatory environment that supports embryo implantation, followed by an immunotolerant phase for fetal development. Trophoblast invasion requires inflammatory activity to invade the uterine cavity via decidual dendritic cells, uterine-natural killer cells, and T helper 1 cells with interleukin (IL)-1, 6 and 8. Estrogen and progesterone are critical for regulating endometrial receptivity [7]. Estrogen induces proliferation and prepares the endometrium, whereas progesterone drives decidualization and maintains the window of implementation. The blastocyst and endometrium secrete various factors (e.g., leukemia inhibitory factor, integrins, and adhesion molecules) to synchronize their development and facilitate implantation [8]. Endometrial glands produce histotrophs to support embryo survival and implantation. Decidualization supports embryo implantation and controls embryo quality. The maternal immune system creates an immunotolerant environment at the maternal-embryo interface via immunoregulatory cells, including T helper 2 cells with IL-10 and regulatory T cells, which are crucial for maintaining tolerance to paternal antigens to maintain fetal development in the cavity after implementation [7].

Chronic hyperglycemia in DM can cause hypothalamic–pituitary–ovarian (HPO) dysfunction with hormone impairment, DNA damage, oxidative and endoplasmic reticulum stress, and mitochondrial dysfunction [9]. High blood sugar causes protein misfolding in the endoplasmic reticulum, which can trigger apoptosis and inflammation [10]. Oxidative stress and inflammation disrupt normal cellular functions and induce damage to placental tissues, increasing trophoblast differentiation and glycogen accumulation and dysregulating glucose metabolism, and angiogenesis affects nutrient and oxygen delivery to the fetus, which are crucial for fetal development.

Pregnancy loss in a hyper-inflammatory state increases the risk of cardiovascular events [11], chronic obstructive pulmonary disease [12] and rheumatoid arthritis [13] within several years. Psychological morbidity is common after pregnancy loss, including increases in the risk of anxiety, depression, posttraumatic stress disorder, and suicide [1]. There is little evidence that pre-pregnancy DM is related to pregnancy loss in the Asian population. We retrospectively utilized data from Taiwan’s National Health Insurance Research Database (NHIRD). By analyzing a large cohort of women of childbearing age, we sought to elucidate the impact of DM on pregnancy outcomes and identify potential comorbid conditions that may increase the risk of pregnancy loss.

This study aimed to evaluate the association between maternal DM and the risk of pregnancy loss in the Chinese population. This study underscores the importance of effective diabetes management and the need for targeted interventions to mitigate the risk of pregnancy loss in women with DM. Therefore, healthcare staff need to develop clinical strategies that can improve maternal and fetal health outcomes, comprehensively manage diabetes, and address associated comorbidities to increase pregnancy viability.

## 2. Materials and Methods

The National Health Insurance system was implemented in 1995 for citizens’ healthcare and covers more than 99.9% of citizens in Taiwan. Each healthcare institution must provide data for the payment of insurance claims every month. The NHIRD is a comprehensive dataset provided by the National Insurance Administration that includes patients’ birthdays, visit dates, disease codes, procedure codes, medication codes and insurance payments [14].

DM patients were identified from the Taiwanese NHIRD by the *International Classification of Diseases*, 9th Revision, Clinical Modification (ICD-9 CM) code 250. The childbearing age of women was defined as between 20 and 50 years. Females of childbearing age who had been diagnosed with DM and had more than 3 visits from 2001–2015 were included in the study group. The exclusion criteria were being male, having fewer than 3 visits, being below or above childbearing age, being diagnosed with DM before 2001, and experiencing pregnancy loss before inclusion. The control group included 4-fold more females who were matched for sex, age, and disease severity according to the Charlson Comorbidity Index (CCI) and index date. The event was pregnancy loss, defined as in ICD-9 CM codes 630–639, which was tracked until 31 December 2015.

**Figure 1:**
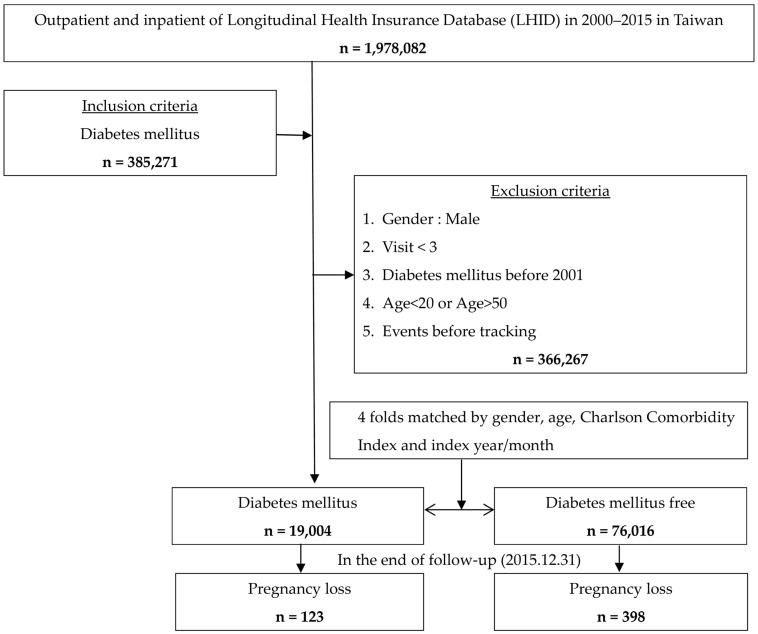
Flowchart of study sample selection from the National Health Insurance Research Database in Taiwan.

Comorbidities were determined by ICD-9-CM codes and included polycystic ovary syndrome (256.4), pelvic inflammatory disease (616), urinary tract infection (599), premenstrual syndrome (625.4), endometriosis (617), autoimmune disease (710, 714), obesity (278), hypertension (401–405), hyperlipidemia (272), chronic kidney disease (580–589), hyperthyroidism (242), fibromyalgia (729.1), chronic fatigue syndrome (780.71), chronic obstructive pulmonary disease (491, 492, 494, 496), asthma (493), alcohol use disorders (291,303, 305.1, 571.0–571.4), depression (296, 300.4, 311), anxiety (300.1–300.3, 300.5–300.9), irritable bowel syndrome (564.1), bladder disorder (596), and polyneuropathy (diabetic: 250.6 and 357.2; other: 729.2). The flowchart of this study is shown in Figure 1. To understand the risk of different types of DM, the ICD-9-CM code of 250.01 was used to define as type 1 DM, and the others (250 except 250.01) were used to define type 2 DM. This study was approved by the Ethics Institutional Review Board of Tri-Service General Hospital (TSGHIRB: B-110-051).

The descriptive statistics, compared between groups for continuous variables, were analyzed with Student’s *t* test, and categorical variables were analyzed with the chi-square test. The significant difference in cumulative incidence was determined via Kaplan–Meier curve with log rank test. Multivariate Cox regression was employed to determine the risk factors associated with pregnancy loss. A *p* value less than 0.05 indicated a significant difference. The analysis was performed with SPSS version 21 (Asia Analytics Taiwan Ltd., Taipei, Taiwan).

## 3. Results

**Figure 2:**
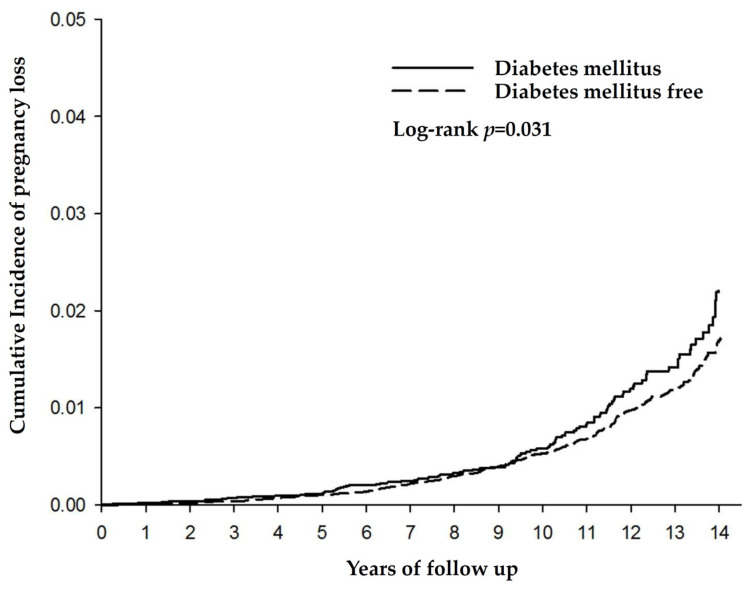
The cumulative incidence of pregnancy loss in the two groups.

There were 19,004 females with DM and 76,016 females without DM included in this study. There were 123 pregnancy losses (cumulative incidence of 2.29%) in the DM group and 398 pregnancy losses (1.7%) in the control group at the 14-year follow-up (*p* = 0.031) (Figure 2). The rate of pregnancy loss was 87.53 per hundred thousand in patients with DM and 71.06 per hundred thousand in patients without DM.

There was a greater prevalence of polycystic ovary syndrome, obesity, hypertension, hyperlipidemia, hyperthyroidism, alcohol use disorder, polyneuropathy, low income and hospital visits in females with DM. Premenstrual syndrome, endometriosis, autoimmune disease, chronic kidney disease, fibromyalgia, chronic obstructive pulmonary disease, asthma, depression, anxiety, irritable bowel disease, and bladder disorders were less common in females with DM (Table 1).

The hazard ratio (HR) for the risk of pregnancy loss in DM patients was 1.407 (95% confidence interval (C.I.): 1.099–1.801, *p* = 0.007). The risk associated with different types of DM had an adjusted HR of 1.498 (95% C.I.: 1.124–1.895, *p* = 0.001) in women with type 2 DM and 1.256 (95% C.I.: 1.058–1.762, *p* = 0.015) in women with type 1 DM. The risk factors were age, with an HR of 1.020 (95% C.I.: 1.004–1.063, *p* = 0.002); pelvic inflammatory disease, with an HR of 2.145 (95% C.I.: 1.911–2.22, *p* < 0.001); premenstrual syndrome, with an HR of 2.067 (95% C.I.: 1.486–2.763, *p* < 0.001); polycystic ovary syndrome, with an HR of 1.792 (95% C.I.: 1.403–2.121, *p* < 0.001); endometriosis, with an HR of 1.42 (95% C.I.: 1.197–1.683, *p* < 0.001); autoimmune disease, with an HR of 1.29 (95% C.I.: 1.111–1.543, *p* < 0.001); urinary tract infection, with an HR of 1.803 (95% C.I.: 1.529–1.88, *p* < 0.001); hyperthyroidism, with an HR of 1.234 (95% C.I.: 1.056–1.503, *p* = 0.001); fibromyalgia, with an HR of 1.353(95% C.I.: 1.204–1.579, *p* < 0.001); chronic fatigue syndrome, with an HR of 1.835 (95% C.I.: 1.106–2.978, *p* < 0.001); asthma, with an HR of 1.382 (95% CI: 1.204–1.595, *p* < 0.001); alcohol use disorder, with an HR of 1.562 (95% C.I.: 1.303–2.701, *p* < 0.001); depression, with an HR of 1.528 (95% C.I.: 1.131–1.735, *p* < 0.001); irritable bowel syndrome, with an HR of 1.340 (95% C.I.: 1.104–1.532, *p* < 0.001); a high CCI score, with an HR of 1.221 (95% C.I.: 1.133–1.468, *p* < 0.001); and fewer visits to medical centers, with an HR of 0.678 (95% C.I.: 0.586–0.917, *p* = 0.001) (Table 2). The risk of pregnancy loss in the first trimester (HR of 1.296 (95% C.I.: 1.089–1.792)) and after the first trimester (HR of 1.724 (95% C.I.: 1.250–1.996)) were noted.

## 4. Discussion

Patients with DM experience a greater rate of pregnancy loss than patients without DM, and patients with DM have a 1.4-fold greater risk of pregnancy loss. Effective management of blood sugar levels is crucial for minimizing these risks and improving pregnancy outcomes for women with DM. Several other conditions also increase the risk of pregnancy loss. Gynecological disorders, autoimmune diseases, mood disorders, irritable bowel syndrome, chronic fatigue syndrome, and fibromyalgia are also risk factors for pregnancy loss, and healthcare professionals need to be aware of these risks and manage patients according to the findings of this study.

A total of 4.9% of these cases occurred in India, which is a developing country [15]. The rate of pregnancy loss was 2.6% in type 1 DM patients and 3.7% in type 2 diabetes patients in New Zealand [16]. Mothers with diabetes mellitus receive more healthcare related to metabolism and obstetrics. DM care programs and pregnancy care programs have been developed for several decades, hence the lower incidence of pregnancy loss in Taiwan. The risk ratio was 1.6 for spontaneous pregnancy loss, whereas HbA1c levels ≥ 5.6% and for risk ratio of 2.1 in every 1 mmol/L increase in fasting blood sugar levels in non-DM mothers were reported [17].

More fetal chromosomal abnormalities occur with increasing maternal age, increasing the likelihood of miscarriage [18]. The most common cause of early pregnancy loss is chromosomal abnormalities in the fetus. A decrease in uterine and hormonal function in older mothers increases the risk of abortion. Older women had a greater risk of abortion than women aged 15–19 years in Ethiopia, with an adjusted OR of 6.13 for women aged 45–49 years [19]. Advanced maternal age showed an adjusted OR of 5.83 in Syrian refugee women [20]. Women who married at more than 26 years of age in Iran were found to have an increased risk of abortion [21]. Our study revealed a 2% increase in pregnancy loss risk with every one-year increase in maternal age.

Polycystic ovary syndrome (POCS) is often associated with insulin resistance, the prevalence of which is increasing in patients with DM. Patients with polycystic ovary syndrome exhibit impaired trophoblast differentiation, increased placental glycogen accumulation, and reduced placental angiogenesis. A mouse study revealed that hyperandrogenism and insulin resistance negatively affect fetal survival through alterations in the mitochondria–ROS–SOD1/Nrf2 axis in the placenta [22]. Our study revealed a 1.8-fold greater risk in women with polycystic ovary syndrome.

Women with endometriosis exhibit a greater inflammatory state. Successful implantation requires a transient increase in proinflammatory cytokines (T helper 1 cells) via immunomodulation, followed by a switch to an anti-inflammatory state (Th2 profile). Recurrent pregnancy loss is linked to immune dysregulation with an increase in proinflammatory cytokines and a decreased anti-inflammatory state [23]. Endometriosis increases the risk of gestational diabetes with an OR of 1.23 [24]. Patients with endometriosis suffer from uncomfortable pain when nonsteroidal anti-inflammatory drugs are used, with an OR of 2.45 spontaneous abortion [25]. The risk of miscarriage in endometriosis had an OR of 1.27 [26]. Our study showed similar results.

DM increased the risk of premenstrual dysphoric disorder (PMDD) in a previous study [27]. Women with PMDD have higher cortisol levels in the late-luteal stage than women without PMDD [28]. Sympathetic overactivity and parasympathetic dysfunction were noted in young females with premenstrual dysphoric syndrome. Depression symptoms decreased the levels of brain-derived neurotrophic factor in a previous study [29]. Sympathetic activity increases inflammation, and parasympathetic dysfunction decreases anti-inflammatory function, increasing the risk of pregnancy loss. Our study revealed an HR of 2.1.

The expression of apoptosis-related Fas and Fas-L and inflammation-related cytokines was seen in the ovaries and uterus in mice treated with lipid polysaccharides [30]. Bacterial lipopolysaccharide induces apoptosis by decreasing Bcl-2 and increasing caspase-3 in the ovaries [31]. Dysbiosis of the vaginal microbiota was related to proinflammatory cytokine levels in a previous study [32]. In our study, women with pelvic inflammatory disease had a 2.1% risk, and those with urinary tract infection had a 1.8% risk of pregnancy loss.

Autoimmune diseases included systemic lupus erythematosus (SLE) and rheumatoid arthritis. Patients with arthritis exhibit sympathetic overactivity with chronic inflammation. There was a greater risk of autoimmune diseases in patients who experienced pregnancy loss with higher proinflammatory cytokine levels with TH1/TH2 imbalance, atherosclerosis, hormone decreases and complement products influencing placental development [7]. Women with Sjögren’s syndrome have a greater risk of pregnancy loss with an RR of 8.85, and of systemic lupus erythematosus with OR 4.90 [33]. Women with rheumatoid arthritis have an increased incidence of PCOS and endometriosis [34]. In a Danish study, women with rheumatoid arthritis had a 1.25-fold increased risk of pregnancy loss [35]. Thyroid antibodies were related to increased risk of gestational diabetes mellitus with a pooled RR of 1.12 [36]. Our study revealed a lower risk of pregnancy loss in women with autoimmune diseases due to the use of regular anti-inflammatory medication.

Hyperthyroidism leads to increased ROS production in mitochondria with oxidative stress [37]. Inadequate treatment for hyperthyroidism in early pregnancy was the main risk factor for pregnancy loss, with an adjusted HR of 1.28 for women who were not treated in early pregnancy and 1.18 for women treated with antithyroid agents in early pregnancy in a Danish population-based study [38]. Our study showed similar results.

Alcohol use during pregnancy is a serious public health issue since it has several detrimental impacts on mothers’ and unborn children’s health. Alcohol consumption during pregnancy is related to the amount of alcohol consumed before pregnancy, the number of pregnancies and a lower education level [39]. The risk of miscarriage was adjusted to an OR of 1.38 for women who consumed alcohol in Ethiopia [19]. Our study revealed a greater risk of pregnancy loss because alcohol use disorder was identified by more severity codes in claims data.

The OR for the risk of miscarriage was 1.25 in patients with depressive disorders in Norway [40]. A higher education level and socioeconomic status and greater stress were associated with an increased risk of abortion in Iran. Serotonin reuptake inhibitors for ant-depressant treatment induce placental insufficiency and increase the risk of miscarriage, with an HR of 1.27 [41]; our study showed similar findings. The discontinuation of treatment before pregnancy was suggested.

Fibromyalgia and chronic fatigue syndrome induce inflammation [42] and increase the risk of pregnancy loss, with an HR of 1.4 in our study. Chronic fatigue syndrome is associated with oxidative stress and immune dysregulation [43,44], and increased the risk of pregnancy loss, with an HR of 1.8 in our study. A mother with asthma has increased chronic inflammation, altered hormonal balance, and medication effects; these factors increase oxidative stress and impair placental function. The risk of pregnancy loss in mothers with asthma had an adjusted HR of 1.21 in Sweden [45]. Our study showed similar results.

The pathophysiology of irritable bowel syndrome involves complex mechanisms, including visceral hypersensitivity and immunoinflammatory disturbances. Inflammation induces the apoptosis pathway, resulting in pregnancy loss. Maternal irritable bowel syndrome is associated with a risk of miscarriage, with an OR of 1.21, and ectopic pregnancy, with an OR of 1.28 [46]. Our study showed similar results. Women with more severe disease and a higher CCI are at greater risk. Our study revealed a lower risk of pregnancy loss, with an HR of 0.7 in women with frequent medical center visits, indicating that better pregnancy care was provided in medical centers. More vigilance is needed regarding the risks associated with diabetes during pregnancy. Good control of DM with HbA1c can reduce adverse pregnancy outcomes [47]. Understanding and addressing the multifaceted risks associated with these conditions can improve pregnancy outcomes for affected women.

There was no significant increase in the risk of pregnancy loss in women with low incomes. High blood sugar levels in diabetic patients also contribute to the accumulation of lipids in the body, leading to hyperlipidemia and obesity. Mothers with pregestational T2DM had greater body weights than mothers without diabetes did in England [48]. The risk of early miscarriage in obese women has an OR of 1.2 [49]., but our study revealed no significant risk of obesity due to the lower prevalence of a body mass index greater than 25 kg/m^2^ in women (23.1 ± 0.24 kg/m^2^ in the 19- to 44-year-old group [50].

There is no significant increase in the risk of hyperlipidemia in the USA [5]. The majority of patients received antilipidemic treatment, which reduced the risk of pregnancy loss in claim data. In Iran, mothers with hypertension had a lower risk of abortion, with an adjusted OR of 0.6, and they received better healthcare to prevent abortion [21]. Our study revealed no significant findings. Abortion increased chronic obstructive pulmonary disease, with an OR of 1.12, with increased inflammation in China [12]. Chronic obstructive pulmonary disease is characterized by increased smoke exposure with hypoxia resulting in lower ovum activity, a lower pregnancy rate and severe symptoms, with asthma attack significantly increasing risk. The OR for the risk of miscarriage was 1.25 in patients with anxiety disorders in Norway [40], but our study revealed no significant difference in the risk of pregnancy loss associated with the lower toxicity of antianxiety agents. More cases of pregnancy loss were induced by bladder disorders, and there was no risk of pregnancy loss. There was no significant risk of chronic kidney disease or polyneuropathy, possibly because renal or peripheral nerve involvement is an end complication of DM with inflammation, and these patients have less pregnancy willingness and pregnancy rates.

In addition to controlling blood sugar, treatments that target oxidative stress, mitochondrial impairment and hyperinflammation reduce DM complications [51]. These findings suggest that managing oxidative stress could be a potential therapeutic approach. Treatment with the antioxidant N-acetylcysteine improved fetal survival in DHT+insulin-treated pregnant rats [22]. The antioxidant and anti-inflammatory effects of melanin reduce diabetic complications [52]. Suitable exercise to increase parasympathetic activity decreases inflammation and reduces pregnancy complications [53]. A Mediterranean diet is associated with a lower risk of preterm birth in Western countries [54]. Vaginal micronized progesterone has an effect on threatened miscarriage, with a risk ratio of 1.03, and increases the live birth rate, with a risk ratio of 1.08 for women with one or more previous miscarriages and early pregnancy bleeding [55]. Insulin use causes insulin receptor overload, reducing FSH and LH secretion [56]. Metformin and insulin can control blood sugar equally but, compared with insulin, metformin has a lower risk of abortion, with an OR of 0.81 [57]. Metformin and glucagon-like peptide-1 (GLP-1) agonists have anti-inflammatory effects and are potential treatments for controlling diabetes in pregnant women [57,58].

There were several limitations in this study. First, the blood sugar control conditions were unavailable in the claims data. The relationship between diabetes control and pregnancy loss is worthy of investigation, and registry studies are needed in the future. Second, smoking status, pregnancy number and body mass index data were not provided in the NHIRD. Smoking increased the OR of pregnancy loss by 1.31 in China [59]. More than three pregnancies are associated with a greater risk of abortion in Iran [21]. A higher body mass index is associated with a greater risk of abortion [21,49], and this relationship needs to be studied in Chinese women. Third, fetal loss was similar in mothers with type 1 DM or type 2 DM in previous studies [16,60]. The risk of pregnancy loss was higher with adjusted HR of 1.5 in women with type 2 DM than in women with type 1 DM. The medications used to treat DM were not surveyed, and neither metformin nor GLP-1 agonists had anti-inflammatory effects. However, further studies are needed to address this gap.

## 5. Conclusions

This study revealed that women with DM have an increased risk of pregnancy loss. It is important to educate and aggressively manage blood sugar in women of childbearing age. Older women with other gynecological disorders have a greater risk of pregnancy loss. Early pregnancy at a young age should be encouraged, and gynecological disorders should be managed. Inflammatory conditions caused by other disorders were noted in this study, and reducing inflammation and oxidative stress could reduce the risk of pregnancy complications.

## Figures and Tables

**Table 1:** Baseline data of women with and without diabetes mellitus.

Variables	Total	Diabetes Mellitus	Without Diabetes Mellitus	*p*
Number	95,020	19,004	76,016	1
Low-income	1294 (1.4%)	383 (2%)	911 (1.2%)	<0.001 *
Polycystic ovary syndrome	2968 (3.1%)	1081 (5.7%)	1887 (2.5%)	<0.001 *
Pelvis inflammation disorder	63,262 (66.6%)	12,238 (64.4%)	51,024 (67.1%)	<0.001 *
Urinary tract infection	31,863 (33.5%)	6460 (34%)	25,403 (33.4%)	0.144
Premenstrual syndrome	2786 (2.9%)	410 (2.2%)	2376 (3.1%)	<0.001 *
Endometriosis	10,925 (11.5%)	2007 (10.6%)	8918 (11.7%)	<0.001 *
Autoimmune disease	9531 (10%)	1163 (6.1%)	8368 (11%)	<0.001 *
Obesity	3321 (3.5%)	1727 (9.1%)	1594 (2.1%)	<0.001 *
Hypertension	17,705 (18.6%)	7565 (39.8%)	10,140 (13.3%)	<0.001 *
Hyperlipidemia	21,161 (22.3%)	9319 (49%)	11,842 (15.6%)	<0.001 *
Chronic kidney disease	7815 (8.2%)	1407 (7.4%)	6408 (8.4%)	<0.001 *
Hyperthyroidism	7583 (8%)	1797 (9.5%)	5786 (7.6%)	<0.001 *
Fibromyalgia	39,543 (41.6%)	7505 (39.5%)	32,038 (42.1%)	<0.001 *
Chronic fatigue syndrome	1345 (1.4%)	277 (1.5%)	1068 (1.4%)	0.5907
Chronic obstructive pulmonary disease	15,567 (16.4%)	2085 (11%)	13,482 (17.7%)	<0.001 *
Asthma	17,032 (17.9%)	2406 (12.7%)	14,626 (19.2%)	<0.001 *
Alcoholic disorder	1658 (1.7%)	431 (2.3%)	1227 (1.6%)	<0.001 *
Depression	10,722 (11.3%)	2006 (10.6%)	8716 (11.5%)	0. 003 *
Anxiety	10,141 (10.7%)	1804 (9.5%)	8337 (11%)	<0.001 *
Irritable bowel disorder	13,057 (13.7%)	2063 (10.9%)	10,994 (14.5%)	<0.001 *
Bladder disorder	2517 (2.6%)	455 (2.4%)	2062 (2.7%)	0.01 *
Polyneuropathies	471 (0.5%)	430 (2.3%)	41 (0.1%)	<0.001 *
Urbanization levels				0.773
1 (The highest)	29,386 (30.9%)	5564 (29.3%)	23,822 (31.3%)	
2	31,769 (33.4%)	6235 (32.8%)	25,534 (33.6%)	
3	26,916 (28.3%)	5600 (29.5%)	21,316 (28%)	
4 (The lowest)	6593 (6.9%)	1546 (8.1%)	5047 (6.6%)	
Missing	356 (0.4%)	59 (0.3%)	297 (0.4%)	
Hospital levels				<0.001 *
Medical center	15,775 (16.6%)	3479 (18.3%)	12,296 (16.2%)	
Regional hospital	18,644 (19.6%)	4851 (25.5%)	13,793 (18.1%)	
Local hospital	14,649 (15.4%)	3901 (20.5%)	10,748 (14.1%)	
Clinic	45,952 (48.4%)	6773 (35.6%)	39,179 (51.5%)	

* *p* < 0.05.

**Table 2:** Risk of pregnancy loss according to multivariate Cox regression.

	Crude Hazard Ratio	*p*	Adjusted Hazard Ratio	*p*
Diabetes mellitus	1.682 (95% C.I.: 1.423–1.971)	<0.001 *	1.407 (95% C.I.: 1.099–1.801)	0.007 *
Age	1.034 (95% C.I.: 1.025–1.073)	<0.01 *	1.020 (95% C.I.: 1.004–1.063)	0.002 *
Post CCI	1.311 (95% C.I.: 1.164–1.571)	<0.001 *	1.221 (95% C.I.: 1.133–1.468)	<0.001 *
Low-income	1.567 (95% C.I.: 0.795–1.798)	0.246	1.268 (95% C.I.: 0.575–1.483)	0.387
Polycystic ovary syndrome	6.765 (95% C.I.: 4.282–8.835)	<0.01 *	1.792 (95% C.I.:1.403–2.121)	<0.001 *
Pelvis inflammation disorder	2.245 (95% C.I.: 2.013–2.498)	<0.001 *	2.145 (95% C.I.: 1.911–2.220)	<0.001 *
Urinary tract infection	1.894 (95% C.I.: 1.625–1.972)	<0.001 *	1.803 (95% C.I.: 1.529–1.880)	<0.001 *
Premenstrual syndrome	2.701 (95% C.I.: 1.941–3.177)	<0.001 *	2.067 (95% C.I.: 1.486–2.763)	<0.001 *
Endometriosis	1.443 (95% C.I.: 1.202–1.694)	<0.001 *	1.420 (95% C.I.: 1.197–1.683)	<0.001 *
Autoimmune disease	1.382 (95% C.I.: 1.125–1.553)	<0.001 *	1.290 (95% C.I.: 1.111–1.543)	<0.001 *
Obesity	1.843 (95% C.I.: 1.354–2.241)	<0.001 *	1.068 (95% C.I.: 0.867–1.798)	0.465
Hypertension	1.006 (95% C.I.: 0.435–1.972)	0.904	0.986 (95% C.I.: 0.421–1.933)	0.804
Hyperlipidemia	1.343 (95% C.I.:0.791–1.597)	0.803	1.146 (95% C.I.:0.682–1.371)	0.201
Chronic kidney disease	1.262 (95% C.I.: 0.679–1.486)	0.797	1.135 (95% C.I.: 0.597–1.337)	0.663
Hyperthyroidism	1.680 (95% C.I.: 1.423–1.986)	<0.001 *	1.234 (95% C.I.: 1.056–1.503)	0.001 *
Fibromyalgia	1.403 (95% C.I.: 1.276–1.688)	<0.001 *	1.353 (95% C.I.: 1.204–1.579)	<0.001 *
Chronic fatigue syndrome	2.561 (95% C.I.: 1.897–3.808)	<0.001 *	1.835 (95% C.I.: 1.106–2.978)	<0.001 *
Chronic obstructive pulmonary disease	1.264 (95% C.I.: 0.925–1.440)	0.703	1.116 (95% C.I.: 0.875–1.438)	0.069
Asthma	1.467 (95% C.I.: 1.303–1.765)	<0.001 *	1.382 (95% C.I.: 1.204–1.595)	<0.001 *
Alcoholic disorder	2.016 (95% C.I.: 1.513–2.897)	<0.001 *	1.562 (95% C.I.: 1.303–2.701)	<0.001 *
Depression	1.680 (95% C.I.: 1.27–1.864)	<0.001 *	1.528 (95% C.I.: 1.131–1.735)	<0.001 *
Anxiety	1.065 (95% C.I.: 0.755–1.279)	0.301	0.972 (95% C.I.: 0.734–1.186)	0.702
Irritable bowel disorder	1.513 (95% C.I.: 1.331–1.792)	<0.001 *	1.340 (95% C.I.: 1.104–1.532)	<0.001 *
Bladder disorder	1.030 (95% C.I.: 0.681–1.156)	0.333	0.972 (95% C.I.: 0.513–1.097)	0.498
Polyneuropathies	0.998 (95% C.I.: 0.425–1.201)	0.505	0.706 (95% C.I.: 0.342–1.084)	0.488
Urbanization level				
1 (The highest)	Reference		Reference	
2	1.098 (95% CI:0.511–1.584)	0.465	1.003 (95% C.I.: 0.408–1.438)	0.565
3	1.145 (95% C.I.: 0.562–1.601)	0.43	1.072 (95% C.I.: 0.492–1.483)	0.551
4 (The lowest)	1.246 (95% C.I.: 0.678–1.65)	0.301	1.125 (95% C.I.: 0.533–1.572)	0.48
Hospital levels				
Medical Center	0.655 (95% C.I.: 0.512–0.834)	<0.001 *	0.687 (95% C.I.: 0.586–0.917)	0.001 *
Regional hospital	0.724 (95% C.I.: 0.409–0.978)	0.014 *	0.782 (95% C.I.: 0.432–1.034)	0.132
Local hospital	0.832 (95% C.I.: 0.335–1.106)	0.505	0.89 (95%C.I.: 0.356–1.297)	0.202
Clinic	Reference		Reference	

* *p* < 0.05; C.I.: confidence interval; CCI: Charlson Comorbidity Index.

## Data Availability

Restrictions apply to the availability of these data. Data were obtained from the National Health Insurance database and are available from the authors with the permission of the National Health Insurance Administration of Taiwan.
